# Morphological comparison of five species of poison dart frogs of the genus *Ranitomeya* (Anura: Dendrobatidae) including the skeleton, the muscle system and inner organs

**DOI:** 10.1371/journal.pone.0171669

**Published:** 2017-02-24

**Authors:** Markus Krings, Benjamin Klein, Markus J. Heneka, Dennis Rödder

**Affiliations:** 1 Department of Herpetology, Zoologisches Forschungsmuseum Alexander Koenig, Bonn, Germany; 2 RJL Micro & Analytic, Karlsdorf-Neuthard, Germany; Universitat Trier, GERMANY

## Abstract

The morphology of larvae stages of most amphibians are often completely different than in adults. Tadpole descriptions have historically been based on external characters like morphometrics, color pattern and oral disc structure. Other papers described anatomical details by the use of dissections. The increase in micro-CT scanning technology provides an opportunity to quantify and describe in detail internal characters like skeleton, musculature and organs. To date, no such tadpole descriptions exist for the well-studied Neotropical poison dart frog genus *Ranitomeya* (Anura: Dendrobatidae). Here we provide descriptions of the internal skeletal, musculature and organ structures of five *Ranitomeya* species and then provide morphological comparisons. Contrary to previous observations, closely related species display several morphological differences. For example, we observed considerable variation in chondrocranial characters, the extent of cranial ossifications, the appearance of some cranial muscles and the arrangement of inner organs. Further studies on the tadpole morphology of more species of *Ranitomeya* and other dendrobatid genera are needed to enable us to understand the complete morphological variation in this group.

## Introduction

The genus *Ranitomeya* (Dendrobatoidea: Dendrobatidae, Bauer, 1988) includes a number of very small species which are specialized on mites, ants, collembolans as well as Coleoptera and Lepidoptera larvae as their main food resources [1]. They are distributed in Amazonia, the Guayana countries, the Andes and additionally westward up to Central America [2,3]. Despite adults comparatively well studied, most tadpole descriptions of *Ranitomeya* spp. contain limited descriptive data and no skeletal and muscular investigations for *Ranitomeya* tadpoles are currently available.

Morphometric data, color patterns and the structure of the oral disc are frequently used for identification and description of tadpoles [2,4,5]. In the recent years, the inclusion of inner morphology is becoming increasingly reported [6–8]. Rapid growth in some anuran larvae is an evolutionary advantage avoiding predation, desiccation and other threats. So the body architecture of the tadpoles of species following this strategy is optimized for efficient food intake and digestion. The chondrocranium is composed of the jaws, the brain capsule and the gill apparatus. It is the attachment point of numerous muscles involved in feeding and respiration [9]. The chondrocranium derives from mesenchymal condensations [10]. In higher vertebrates it ossifies to form the skull of adults [11]. Chondrocranial morphology is highly variable [12–14] even among closely related taxa [15,16]. Furthermore, it is associated to the ecology of the respected species [11,15]. Skull development is an important character for the evolution of vertebrates [17,18] and chondrocranial characters were used for phylogenetic studies especially in anurans [19–22]. The body cavity is dominated by the digestive tract [9] and the inner organs of tadpoles are known to be variable in form and shape [23].

The aim of this study is a comparison of inner morphology of different members of the genus *Ranitomeya*. We decided to include the chondrocranium, cranial muscle systems and the inner organs in our morphological comparison.

We provide extensive descriptions of internal morphology of five species of *Ranitomeya*: *R*. *vanzolinii* (Myers, 1982), *R*. *imitator* (Schulte, 1999) *R*. *reticulata* (Boulenger, 1984), *R*. *benedicta* (Brown et al., 2008), *R*. *amazonica* (Schulte, 1999). These five species analyzed in this study are quite distributed over a species tree of this group [2] and characterize the major variation in this genus. *Ranitomeya vanzolinii* and *R*. *imitator* are closely related and both part of the *vanzolinii* species group. *R*. *amazonica* is a representative of the *variabilis* group. *R*. *reticulata* and *R*. *benedicta* represent the *reticulata* species group. Members of the *defleri* species group were not available. We provide a full morphological description for one species and present deviations from this bauplan for the other taxa.

## Material and methods

All tadpoles analyzed in this study were from the collection of the Museum Koenig. They were fixed in PFA and stored in 80% ethanol. All specimens were in Gosner stage 41, the last prometamorphic stage. We used stage 41 tadpoles to find all prometamorphic muscles of the species present, but we like to remind the reader that Gosner stage 41 may also cause some problems: Gosner’s staging table is a rather crude categorization and the rapid remodeling of tadpole anatomy in the following metamorphic stages 42–46 could influence the description. From all species one specimen was critical point dried and served for a micro-CT scan, one was cleared and stained, one was dissected and another one served as voucher specimen (Table 1). For additional measurements further scans were conducted with the dissection and voucher specimens using a slight and reversible iodine staining (0.1% IKI in water for a few hours [24]). All specimens were housed in the collection of the museum Koenig (ZFMK 97357–97369, ZFMK 97375–97379) after analysis. Just the dissected specimen of *R*. *vanzolinii* was extremely damaged and therefore not returned. The exception is *R*. *benedicta* as for this species just three tadpoles were available and no untreated specimen served as voucher specimen.

**Table 1:** Specimens used in this study and there collection numbers.

Species	CT-Scan	Cleared and stained	Dissection	Untreated
*R*. *vanzolinii*	ZFMK 97369	ZFMK 97379		ZFMK 97361
*R*. *imitator*	ZFMK 97368	ZFMK 97377	ZFMK 97364	ZFMK 97358
*R*. *amazonica*	ZFMK 97366	ZFMK 97375	ZFMK 97362	ZFMK 97357
*R*. *benedicta*	ZFMK 97367	ZFMK 97376	ZFMK 97363	
*R*. *reticulata*	ZFMK 97360	ZFMK 97378	ZFMK 97365	ZFMK 97359

The table shows all specimens used for this study with their collection numbers. The columns represent the investigation methods.

For clearing and staining the general procedure as proposed by [25] was modified for tadpoles. Cartilage was stained first by use of Alcian blue. Subsequently the specimens were skinned and inner organs were removed. The tadpoles were bleached with H_2_O_2_ and soft tissue was digested with trypsin. Finally, bony tissue was stained with Alizarin red.

Micro-CT scans were conducted using a SkyScan 1272 and SkyScan 1172 (Bruker microCT, Kontich, Belgium). The source voltage was 40–70 kV. The resolution of the scans used for reconstructing the chondrocranium, cranial muscles and ossification was between 1.75 μm and 3.5 μm. The resolution of the scans used for additional measurement was 13.19 μm. Raw data was reconstructed with NRecon (Version 1.6.10, Bruker microCT, SkyScan, Kontich, Belgium). 3D-Models (Boissonat surface) were built in Reconstruct (Version 1.1.0.0, SynapseWeb, [26]) by tracing the profiles of chondrocranial structures in the section images. Finally, we smoothed the models and produced pictures in Blender (Version 2.75, Blender Foundation, Amsterdam, Netherlands). 3D pdfs were produced in Simlab (Version 7.1.1, Simulation Lab Software L.L.C.) after smoothing (Taubin Smooth, λ = 0.5, μ = -0.53, up to two steps) the models and reducing triangles (Quadric Edge Collapse Decimation) in Meshlab (Version 1.3.3, Visual Computing Lab–ISTI—CNR).

The chondrocranium of the tadpoles was reconstructed from the CT scans and ossifications in the chondrocranium were identified and visualized. Moreover, chondrocranial muscles were reconstructed from the CT-scans. We would like to remind the reader that the 3D pdfs (S1–S4 3D pdfs) only show reconstructions of the real cranial characters and may be affected by artifacts. Therefore, a dissection was performed to support the CT results on cranial muscles and to show the main inner organs of the *Ranitomeya* tadpoles. Results on hard tissue morphology were supported by cleared and stained specimens. Concerning the cartilage the terms “fused to”, “merges into” and “diverges from” are used for a fluent continuation of the cartilage. The terms “in contact to” and “touch” describe a secondary symphysis of two neighboring cartilaginous structures.

Concerning nomenclature of the different structures in the chondrocranium and of the cranial muscles we follow predominantly Haas [19,20,27] as well as Haas et al. [7] for three reasons: The publications of Haas are the most recent and most modern descriptions of tadpole morphology. Moreover, Haas et al. [7] is the only paper using micro-CT scans for tadpole descriptions, which is comparable to the methods used in this study. Lastly, the terminology of Haas is based on widely accepted and frequently cited basic morphological papers [28–32]. Concerning the inner organs we used Viertel and Richter [33] and Sanchez [23] as main references.

**Fig 1:**
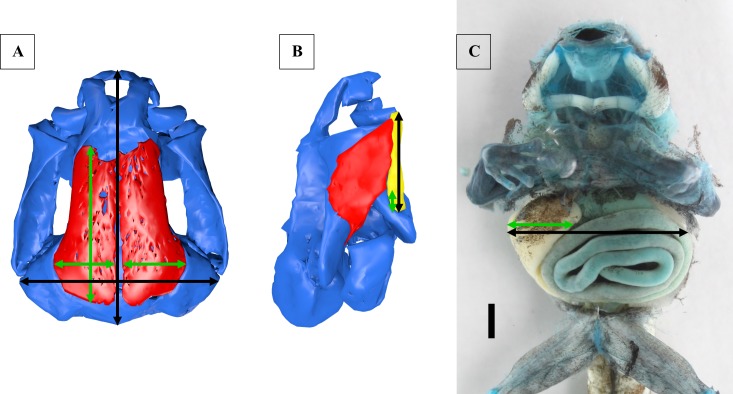
Anatomical measurements. (A) Chondrocranium and frontoparietalia of *R*. *amazonica* in a dorsal view. Length and width of the frontoparietalia are marked by green double arrows, length and width of the chondrocranium (without palatoquadratum) are marked by black double arrows. (B) Chondrocranium (blue), *m*. *suspensorioangularis* (red) and *m*. *hyoangularis* (yellow) of *R*. *imitator* in a lateral view. The length of the *m*. *hyoangularis* is marked by a black double arrow. The part running without association to the *m*. *suspensorioangularis* is marked by a green double arrow. (C) Dissection of *R*. *amazonica* in a ventral view, scale bar 1mm. The visible part of the liver at the widest point of the inner organs is marked by a green double arrow. The complete width of all inner organs at this point is marked by a black double arrow.

We measured characters of the tadpoles that appeared to be different among the species. Because the tadpoles differed in size, we used relative instead of absolute measurements. We measured the frontoparietalia (maximum length multiplied with maximum width) in relation to the chondrocranium (complete length multiplied with maximum width at otic capsules, Fig 1A). The distance the *m*. *hyoangularis* runs without contact to the *m*. *suspensorioangularis* was measured and divided by the total length of the *m*. *hyoangularis* (Fig 1B). Furthermore we measured the maximum width of the inner organs and the percentage that is covered by the liver (Fig 1C).

## Results and discussion

For simplicity, we provide a full description of stage 41 tadpoles of *R*. *vanzolinii* and present only deviations from this general bauplan for the stage 41 tadpoles of other species. The description of the cranial muscles (Table 2) and inner organs is mainly based on the micro-CT scan and the dissection. The chondrocranium and cranial ossifications are described by use of the micro-CT scan and the cleared and stained specimen.

**Table 2:** Origin and insertion of chondrocranial muscles in a stage 41 tadpole of *R*. *vanzolinii*.

Musculus	Origin	Insertion
Lev. mand. long. superf.	Curvatura posterior quadrati	Cartilago meckeli
Lev. mand. long. prof.	Curvatura posterior quadrati	Cartilago suprarostralis
Lev. mand. ext. superf.	Absent	
Lev. mand. ext. prof.	Inner side of palatoquadratum	Cartilago suprarostralis
Lev. mand. art.	Inner side of palatoquadratum	Cartilago meckeli
Lev. mand. int.	Ventral capsula auditiva	Cartilago meckeli
Lev. mand. lat.	Absent in the studied stage	
Quadratoangularis	Ventral palatoquadratum	Retroarticular process
Hyoangularis	Ventrolateral ceratohyale	Retroarticular process
Suspensorioangularis	Palatoquadratum	Retroarticular process
Orbitohyoideus	Processus muscularis	Posteroventral ceratohyale
Suspensoriohyoideus	Dorsolateral palatoquadratum	Posteroventral ceratohyale
Submentalis	Ventral cartilago infrarostralis	Arching from one side to the other
Mandibulolabialis	Cartilago meckeli	Lateral oral disc
Intermandibularis	Median raphe	Cartilago meckeli
Interhyoideus	Median raphe	Ventral ceratohyale
Geniohyoideus	Ventral planum hypobranchiale	Cartilago infrarostralis / soft tissue of glottis
Subarc. obl. II	Processus urobranchialis	Ceratobranchiale III
Subarc. rect. I (dorsal)	Processus posterior hyalis	Ceratobranchiale I
Subarc. rect. I (ventral)	Processus posterior hyalis	Ceratobranchiale III
Subarc. rect. II-IV	Basal ceratobranchiale III or IV	Ceratobranchiale II
Rectus cervicis	Abdominal wall (as m. rectus abdominis)	Ceratobranchiale III
Constr. branch. I	Absent	
Constr. branch. II	Basal ceratobranchiale II	Commissura terminalis I-II
Constr. branch. III	Basal ceratobranchiale III	Terminal ceratobranchiale II
Constr. branch. IV	Ceratobranchiale III	Terminal ceratobranchiale III
Lev. arc. branch. I	?	?
Lev. arc. branch. II	?	?
Lev. arc. branch. III	Lateral capsula auditiva	Commissura terminalis II-III
Lev. arc. branch. IV	Lateral capsula auditiva	Posterior ceratobranchiale IV
Tympanopharyngeus	Posterior capsula auditiva	Oesophagal and pericardial soft tissue
Interhyoideus posterior	?	?
Diaphragmatopraecordialis	?	?
Diaphragmatobranchialis	Abdominal wall	Commissura terminalis III-IV

For a more specific table comparing all species analyzed in this study see S1 Table.

### Ranitomeya vanzolinii

#### Chondrocranium

**Fig 2:**
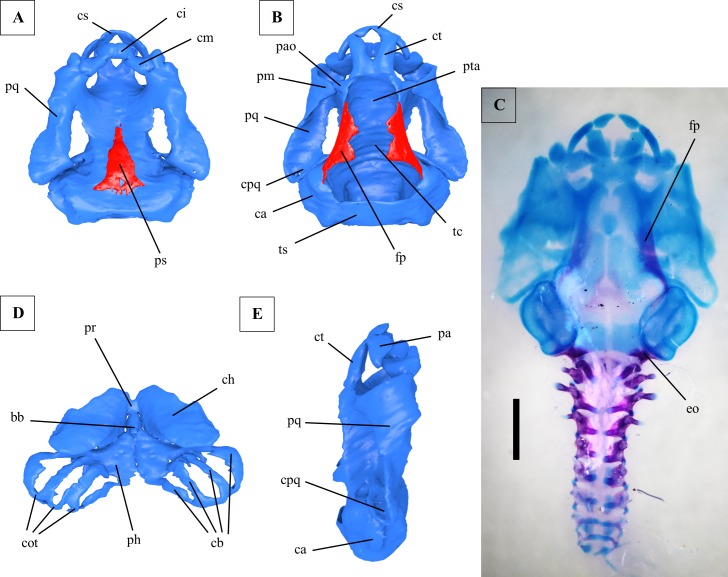
Larval chondrocranium of *Ranitomeya vanzolinii*. (A) Ventral view, ossifications shown in red, cartilage in blue. (B) Dorsal view, ossifications shown in red, cartilage in blue. (C) Dorsal view, cleared and stained specimen (cranium and backbone only), ossifications stained red, cartilage blue, scale bar 1mm. Limbs and girdles were lost during clearing. (D) Hyobranchial apparatus. (E) Lateral view. A, B, D and E were reconstructed from ZFMK 97369. C shows ZFMK 97379. All tadpoles used were in Gosner stage 41. bb: basibranchiale, ca: capsula auditiva, cb: ceratobranchialia, ch: ceratohyale, ci: cartilago infrarostralis, cm: cartilago meckeli, cot: commissurae terminales, cpq: curvatura posterior quadrati, cs: cartilago suprarostralis, ct: cornu trabeculae, eo: exoccipitale, fp: frontoparietale, pa: pars alaris, pao: processus antorbitalis, ph: planum hypobranchiale, pm: processus muscularis, pq: palatoquadratum, pr: pars reuniens, ps: parasphenoid, pta: planum trabeculae anticum, tc: trabecula cranii, ts: tectum synoticum.

The upper jaw is formed by the cartilago suprarostralis (Fig 2A and 2B). Left and right side of the cartilago suprarostralis are separated by a symphysis. An additional symphysis separates a lateral part (pars alaris, Fig 2E). Dorsal to the latter symphysis the cornu trabeculae (Fig 2B and 2E) touches the cartilago suprarostralis. The two cornua are slender in shape. Caudally they merge into the unpaired planum trabeculae anticum (Fig 2B). Lateral to the planum the processus antorbitalis (Fig 2B) points towards the processus muscularis of the palatoquadratum. Posterior to the processus antorbitalis the cartilago orbitalis borders the braincase. Numerous foramina for cranial nerves and arteries in the braincase were found but are not reconstructed. The trabecula cranii is the caudalmost part of the basicranium (also including cornu trabeculae and planum trabeculae anticum, Fig 2B) and is edged by the auditory capsules (capsulae auditivae, Fig 2B and 2E). The capsules are dorsally connected by the tectum synoticum (Fig 2B). The auditory capsules show a lateral crista parotica. The foramen ovale is located ventral to the crista (not reconstructed). Laterally the chondrocranium is dominated by a big and massive palatoquadratum (Fig 2A, 2B and 2E). Anteriorly it is fused to the basicranium by the commissura quadrato-cranialis anterior, posteriorly by a small ascending process. The caudal end of the palatoquadratum is formed by the curvatura posterior quadrati (Fig 2B and 2E), which touches the capsula auditiva.

The lower jaw is formed by the cartilago infrarostralis (Fig 2A), which laterally touches the paired cartilago meckeli (Fig 2A). The cartilago infrarostralis is divided by a median symphysis. The cartilago meckeli forms the processus retroarticularis and touches the anteroventral part of the palatoquadratum. Posterior to the lower jaw cartilage the hyobranchial apparatus is located. It consists of the paired ceratohyale (Fig 2D) flanking the unpaired basibranchiale (Fig 2D) with its processus urobranchialis. Ceratohyalia and basibranchiale touch each other in the so called pars reuniens (Fig 2D). The condylus of the ceratohyale touches the palatoquadratum. The posterolateral part of the ceratohyale is called processus lateralis hyalis and partly covers the first gill arch in a ventral view. Posteromedially it additionally forms the processus posterior hyalis. The basibranchiale and the ceratohyale touch the planum hypobranchiale, which is located posterior to them. Four gill arches (ceratobranchialia I-IV) diverge from the planum (Fig 2D), which is clearly separated into two parts by a medial cleft. The first gill arch forms the processus anterior branchialis at its anterior ridge. The gill arches (Fig 2D) fuse posteriorly in terminal commissures (commissurae terminales, Fig 2D) forming a completed gill basket. Numerous gill rakers are present at the ceratobranchialia (not reconstructed).

A prominent bone at the chondrocranium is the paired frontoparietale (Fig 2B), because it is superficially visible in a dorsal view. It does not completely cover the braincase but is a lateral ossification. The prootic is an endochondral ossification in the anteromedial wall of the capsula auditiva. The exoccipital is an endochondral ossification in the posterior wall of the capsula auditiva. The unpaired parasphenoid (Fig 2A) is located ventral to the planum trabeculae anticum and the trabecula cranii (in combination also called planum basale).

#### Musculi levatores mandibulae

**Fig 3:**
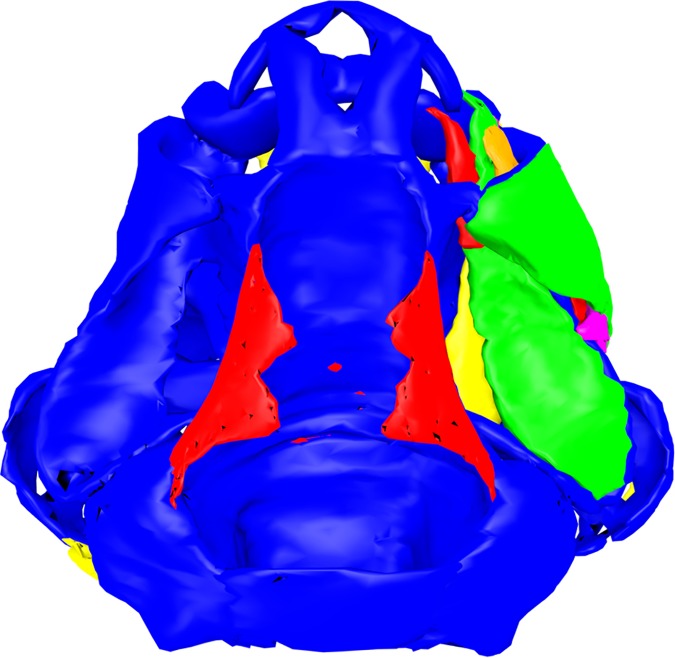
Chondrocranial muscles of *Ranitomeya vanzolini*. The chondrocranium (blue) is shown in a dorsal view with all cranial muscles. The reconstruction was done from a stage 41 tadpole (ZFMK 97369). For further information on the muscles we provide a 3D pdf in S1 3D pdf. In this model all muscles can be added to or removed from the scene by a checkbox. The model is rotatable and can be seen in all perspectives.

The *m*. *levator mandibulae longus* (Fig 3 and S1 3D pdf) originates broadly and fleshy on the dorsoposterior part of the palatoquadrate. A distinction of *m*. *levator mandibulae longus superficialis* and *profundus* at the origin is not possible. Posterior to the processus muscularis and its connection to the processus antorbitalis the two parts of *m*. *levator mandibulae longus* separate into the *m*. *lev*. *mand*. *longus superficialis* dorsomedially and *profundus* ventrolaterally (Fig 3 and S1 3D pdf). The *m*. *lev*. *mand*. *longus superficialis* then inserts at the dorsoposterior part of the cartilago meckeli while the *m*. *lev*. *mand*. *longus profundus* reduces to a tendon and inserts ventrolaterally at the lateral suprarostral cartilage (pars alaris). The *m*. *lev*. *mand*. *externus profundus* (Fig 3 and S1 3D pdf) originates fleshy at the inner side of the processus muscularis. On its way to the pars alaris it fuses with the *m*. *lev*. *mand*. *longus profundus* (*superficialis* and *profundus* are already clearly separated here). The *m*. *lev*. *mand*. *externus superficialis* is absent. The *m*. *lev*. *mand*. *articularis* (S1 3D pdf) originates at the inner side of processus muscularis, ventral to the *m*. *lev*. *mand*. *externus*. It inserts at the cartilago meckeli, lateral to the *m*. *lev*. *mand*. *longus superficialis*. The *m*. *lev*. *mand*. *internus* (Fig 3 and S1 3D pdf) originates fleshy at the ventral part of the otic capsule at about the same depth as the *m*. *lev*. *mand*. *longus*. It continuously reduces in diameter until the processus muscularis and the division of *mm*. *lev*. *mand*. *longus superficialis* and *profundus*. Then it crosses all other muscles of the levator mandibulae complex ventrally as a long tendon and inserts at the cartilago meckeli, lateral to all other muscles of the complex. The *m*. *lev*. *mand*. *lateralis* was not found in our tadpoles. Haas [20] mentions, that this muscle develops shortly before metamorphosis in some species. For these reasons we code it as “absent in the studied stage”.

#### Angularis group

The *m*. *quadratoangularis* (S1 3D pdf) originates ventrally on the palatoquadrate close to its connection to the ceratohyale. It inserts at the retroarticular process, ventrally at the cartilago meckeli. Despite its insertion point it is completely covered by the other muscles of the angularis group. The *m*. *hyoangularis* (S1 3D pdf) originates fleshy at the ventrolateral aspect of the ceratohyale. It runs ventrolaterally to *m*. *quadratoangularis* but inserts slightly dorsoposteriorly at processus retroarticularis. The *m*. *suspesorioangularis* (S1 3D pdf) originates fleshy on the dorsal and posterolateral aspect of the palatoquadrate. At the origin the fibers built two portions for a short distance. Additionally some fibers of the ventromedial portion originate from the soft tissue borderline of the alimentary canal. The *m*. *suspesorioangularis* inserts slightly posterolateral to the *m*. *hyoangularis* at the processus retroarticularis. On their way to insertion the *m*. *hyoangularis* and the *m*. *suspensorioangularis* meet and run in close association.

#### *Musculus orbitohyoideus* and *musculus suspensoriohyoideus*

The *m*. *orbitohyoideus* (Fig 3, S1 3D pdf) originates broadly and fleshy on the processus muscularis of the palatoquadrate. It is a big muscle that runs laterally towards the ceratohyale covering big parts of the *m*. *suspensorioangularis* and the origin of *m*. *hyoangularis*. Its insertion at the posteroventral part of the ceratohyale is also broad and fleshy. Shortly before this insertion the *m*. *suspensoriohyoideus* (S1 3D pdf) fuses with the *m*. *orbitohyoideus*. The fibers of the *m*. *suspensoriohyoideus* might insert some more laterally than most fibers of the *m*. *orbitohyoideus*. The origin of the *m*. *suspensoriohyoideus* is dorsolaterally on the palatoquadrate. The origin is much smaller and clearly posterior to the origin of the *m*. *orbitohyoideus*.

#### *Musculus submentalis* and *musculus mandibulolabialis*

**Fig 4:**
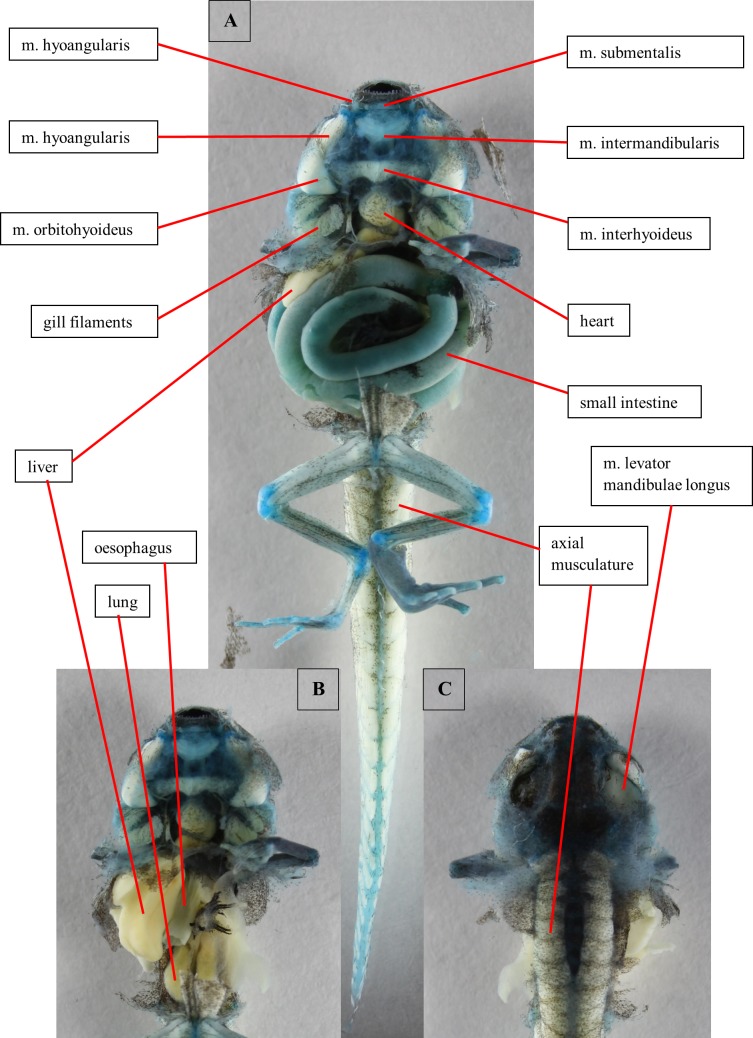
Dissection of a tadpole of *Ranitomeya vanzolinii*. Dissection of a stage 41 tadpole. Cartilage is stained with alcian blue. (A) Ventral view, body cavity opened, skin removed. (B) Ventral view, intestine removed. (C) Dorsal view, eyes removed. Scale bars 1mm.

The *m*. *submentalis* is an unpaired ventral muscle that originates from the ventral cartilago infrarostralis. It arches from one side to the other. The *m*. *mandibulolabialis* originates from the cartilago meckeli and inserts on the lateral oral disc. Both muscles were identified in the dissection (Fig 4) but could not be reconstructed from the CT scans.

#### *Musculus intermandibularis* and *musculus interhyoideus*

The *m*. *intermandibularis* (S1 3D pdf) inserts on the anteroventral surface of the cartilago meckeli and arches from one side to the other. It extends far posteriorly covering the anterior part of the ceratohyale in a ventral view. The *m*. *interhyoideus* (S1 3D pdf) inserts ventrally on the ceratohyale and also arches from one side to the other covering the basibranchiale in a ventral view. Both muscles originate from a median raphe.

#### *Musculus geniohyoideus*, *musculus subarcualis obliquus II*, *musculus subarcualis rectus I*, *musculus subarcualis rectus II-IV* and *musculus rectus cervicis*

The *m*. *geniohyoideus* (S1 3D pdf) originates fleshy on the ventral surface of the planum hypobranchiale close to the third gill arch. It runs over the ventral surface of the hyobranchial apparatus as a slender muscle. Moreover, it reduces in diameter on its way to the insertion on the ventral aspect of the cartilago infrarostralis. Posterior to the cartilago infrarostralis the medial portion of the *m*. *geniohyoideus* separates and inserts on the soft tissue of the glottis (larval tongue). The *m*. *subarcualis obliquus II* (S1 3D pdf) originates as a tendon from the posterior end of the basibranchiale (processus urobranchialis). It runs more ventrally than the *m*. *geniohyoideus* and inserts on the third gill arch, close to the insertions of the *m*. *subarcualis rectus I* (ventral portion) and the *m*. *rectus cervicis*. The *m*. *subarcualis rectus I* (S1 3D pdf) cannot be completely divided into two portions. It originates on the lateral surface of the ceratohyale (processus posterior hyalis). In a lateral view the origin is largely covered by the processus lateralis hyalis. Posterior to the origin it splits into two fiber bundles. The dorsal portion inserts on the first gill arch, the ventral one on the third gill arch. The origin of the *m*. *subarcualis rectus II-IV* is difficult to locate. It may be on the ceratobranchiale III or on the ceratobranchiale IV. Maybe fibers originate from both gill arches. The muscle inserts on the ceratobranchiale II. Because of its uncertain origin this muscle is not shown in the 3D reconstruction. The *m*. *rectus cervicis* (S1 3D pdf) inserts on the third gill arch very close to the insertion of the *m*. *subarcualis rectus I* (ventral portion). It continues as the *m*. *rectus abdominis* which originates broadly from the abdominal wall ventral to the liver and the intestine (not reconstructed).

#### Branchial constrictors and levators and *musculus tympanopharyngeus*

Branchial constrictors are hard to identify among numerous gill filaments. The *m*. *constrictor branchialis I* sensu Haas [19] could not be found. The *m*. *constr*. *branch*. *II* (S1 3D pdf) was found originating on the base of the ceratobranchiale II, running close to the first gill arch and finally inserting on the terminal commissure of ceratobranchialia I and II. Identification of *m*. *constr*. *branch*. *III* and *IV* was complicated. The *m*. *constr*. *branch*. *III* (S1 3D pdf) originates on the basal part of ceratobranchiale III extremely close to the insertion of the *m*. *subarcualis rectus I*. It inserts at the very end of the ceratobranchiale II directly before its terminal commissure with the ceratobranchiale III. The *m*. *constr*. *branch*. *IV* (S1 3D pdf) originates on the ceratobranchiale III as well but posterior to the origin of the *m*. *constr*. *branch*. *III*. It inserts on the posterior part of ceratobranchiale III. Identification of branchial levators finally worked for *mm*. *levatores arcuum branchialium III* and *IV*. The *mm*. *lev*. *arc*. *branch*. *I* and *II* might be there but cannot be reconstructed. A tiny layer of soft tissue can be seen at the estimated way of the *mm*. *lev*. *arc*. *branch*. *I* and *II* but an identification of these muscles is impossible. If they are present they must be very small fiber bundles. The *m*. *lev*. *arc*. *branch*. *III* and *IV* (S1 3D pdf) originate commonly from the capsula auditiva. The *m*. *lev*. *arc*. *branch*. *III* inserts at the terminal commissure of the ceratobranchialia II and III but closer to the ceratobranchiale II. The *m*. *lev*. *arc*. *branch*. *IV* inserts on the posterior ceratobranchiale IV. The *m*. *tympanopharyngeus* (S1 3D pdf) also originates from the capsula auditiva but posterior to the branchial levators. It runs close to the *m*. *lev*. *arc*. *branch*. *IV* and inserts on oesophagal and pericardial soft tissue (not reconstructed).

#### *Musculus interhyoideus posterior*, *musculus diaphragmatopraecordialis* and *musculus diaphragmatobranchialis*

The *m*. *interhyoideus* posterior and the *m*. *diaphragmatopraecordialis* could not be identified. These muscles often consist of few, loosely spaced fibers encircling the peribranchial chamber. They are hardly to identify in CT scans and in dissections they often get lost during skinning [20]. The *m*. *diaphragmatobranchialis* was found to originate from the abdominal wall and to insert on the commisura terminalis of the ceratobranchialia III and IV. It was difficult to identify on the CT scans and is not shown in the 3D reconstructions.

#### Alimentary canal and inner organs

The oesophagus is first concealed by the intestine (Fig 4). Than it does a U-turn in between the liver lobes. Here it passes into the manicotto glandulare, the larval stomach. The exact transition point cannot be localized because the oesophagus and the manicotto do not differ in the CT images and in the visual dissection. These two sections build the foregut. The foregut can be clearly separated from the following intestinal parts of the digestive canal by the structure of its epithelium. The foregut epithelium is composed of columnar cells. The epithelium of the intestine is smooth, no cellular structure is visible. The intestine is coiled sinistrally (ventral view), but coiling direction changes after few loops (Fig 4). On the right body side the liver (Fig 4) and the associated gall bladder are found. In a ventral view of the opened body cavity just a slender part of the liver is visible anteriorly. The rest is concealed by the intestine (Fig 4). Furthermore, the pancreas is found left to the oesophagus, centrally in the body cavity. Dorsally the larval lungs are located (Fig 4). They are hard to distinguish from the axial musculature because they are situated directly ventral to the vertebral column.

### Ranitomeya imitator

**Fig 5:**
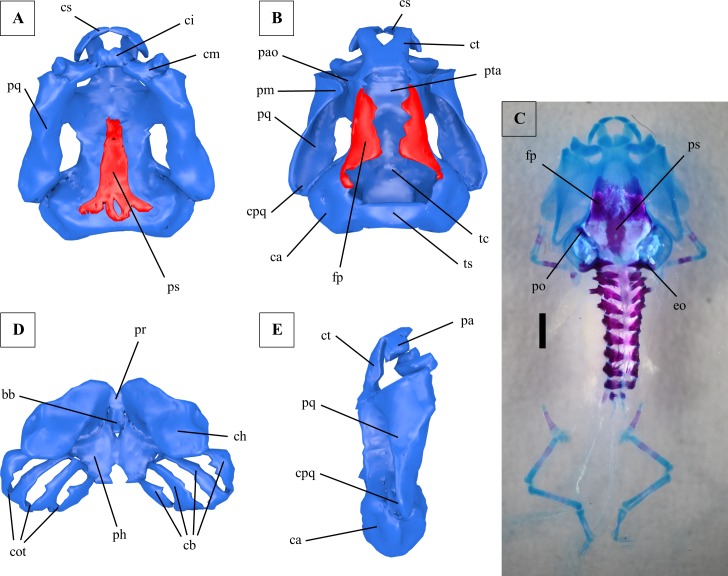
Larval chondrocranium of *Ranitomeya imitator*. (A) Ventral view, ossifications shown in red, cartilage in blue. (B) Dorsal view, ossifications shown in red, cartilage in blue. (C) Dorsal view, cleared and stained specimen, ossifications stained red, cartilage blue, scale bar 1mm. (D) Hyobranchial apparatus. (E) Lateral view. A, B, D and E were reconstructed from ZFMK 97368. C shows ZFMK 97377. All tadpoles used were in Gosner stage 41. For abbreviations see Fig 2, additionally: po: prootic.

**Fig 6:**
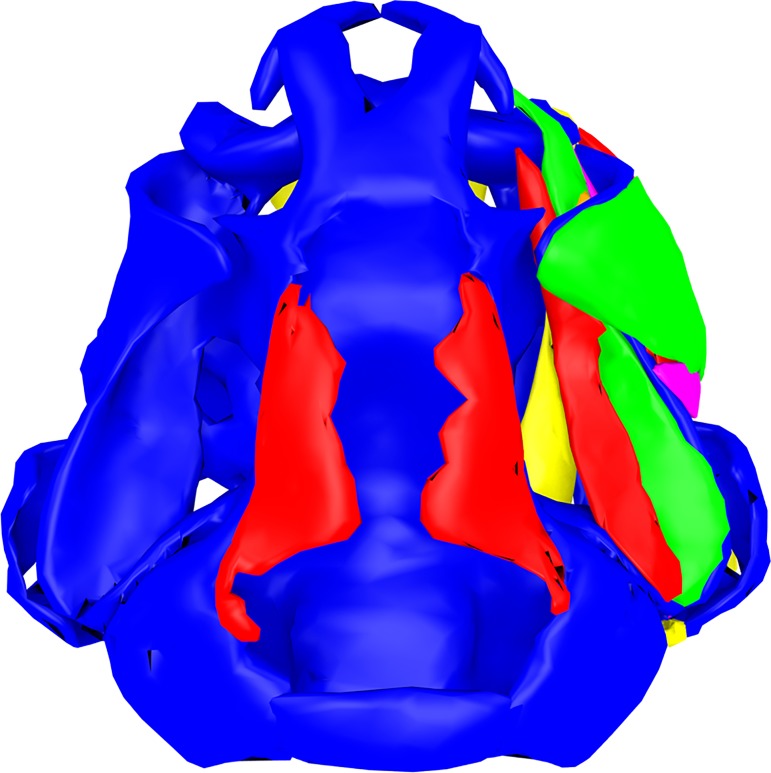
Chondrocranial muscles of *Ranitomeya imitator*. The chondrocranium (blue) is shown in a dorsal view with all cranial muscles. The reconstruction was done from a stage 41 tadpole (ZFMK 97368). For further information on the muscles we provide a 3D pdf in S2 3D pdf. In this model all muscles can be added to or removed from the scene by a checkbox. The model is rotatable and can be seen in all perspectives.

**Fig 7:**
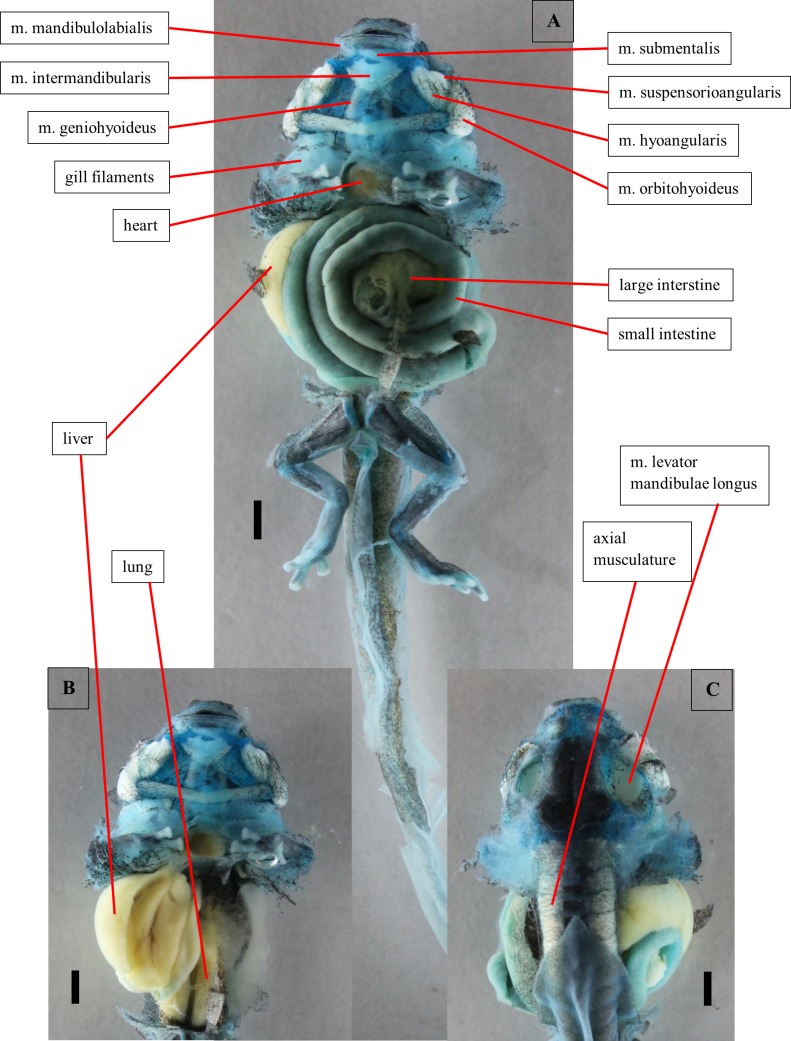
Dissection of a tadpole of *Ranitomeya imitator*. Dissection of a stage 41 tadpole (ZFMK 97364). Cartilage is stained with alcian blue. (A) Ventral view, body cavity opened, skin removed. (B) Ventral view, intestine removed. (C) Dorsal view, eyes removed. Scale bars 1mm.

The frontoparietals of *R*. *imitator* are bigger in their extension than in *R*. *vanzolinii* (Fig 5B and 5C). In *R*. *imitator* the origins of *mm*. *levator mandibulae longus superficialis* and *profundus* could be distinguished. Nevertheless the two muscles originate side by side on the curvatura posterior quadrati (Fig 6, S2 3D pdf). The *m*. *suspensorioangularis* originates with three heads in *R*. *imitator*. Two fiber bundles originate from the dorsal and posterolateral palatoquadratum, which is also the origin of this muscle in *R*. *vanzolinii*. A third head originates from the alimentary canal (S2 3D pdf). The two portions of the *m*. *subarcualis rectus I* have no common origin in *R*. *imitator* (S2 3D pdf). The *mm*. *levatores arcuum branchialium III* and *IV* can be also told apart at their origin (S2 3D pdf). In *R*. *imitator* the *m*. *tympanopharyngeus* originates posteroventral to the *mm*. *lev*. *arc*. *branch*. *III* and *IV* (S2 3D pdf). The *m*. *diaphragmatobranchialis* could not be identified in *R*. *imitator*. The liver of *R*. *imitator* is less concealed by the intestine in a ventral view. The lower liver lobe is superficially visible (Fig 7).

### Ranitomeya amazonica

**Fig 8:**
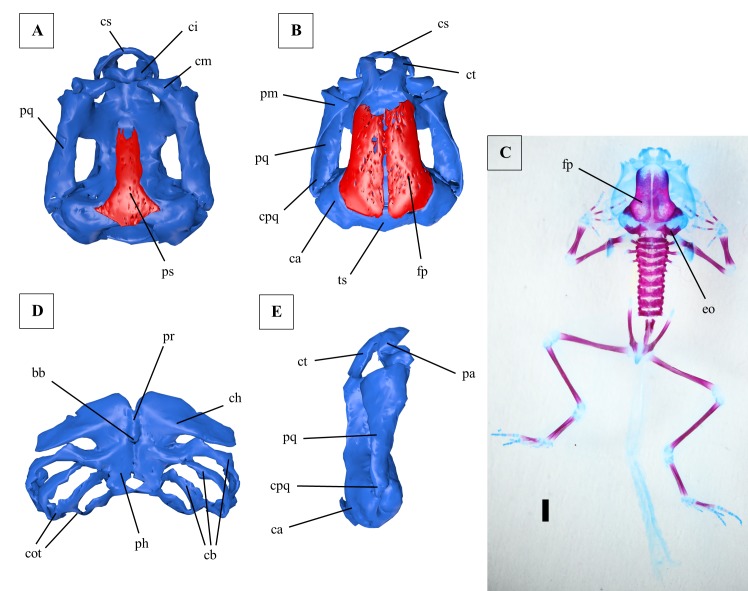
Larval chondrocranium of *Ranitomeya amazonica*. (A) Ventral view, ossifications shown in red, cartilage in blue. (B) Dorsal view, ossifications shown in red, cartilage in blue. (C) Dorsal view, cleared and stained specimen, ossifications stained red, cartilage blue, scale bar 1mm. (D) Hyobranchial apparatus. (E) Lateral view. A, B, D and E were reconstructed from ZFMK 97366. C shows ZFMK 97375. All tadpoles used were in Gosner stage 41. For abbreviations see Fig 2.

**Fig 9:**
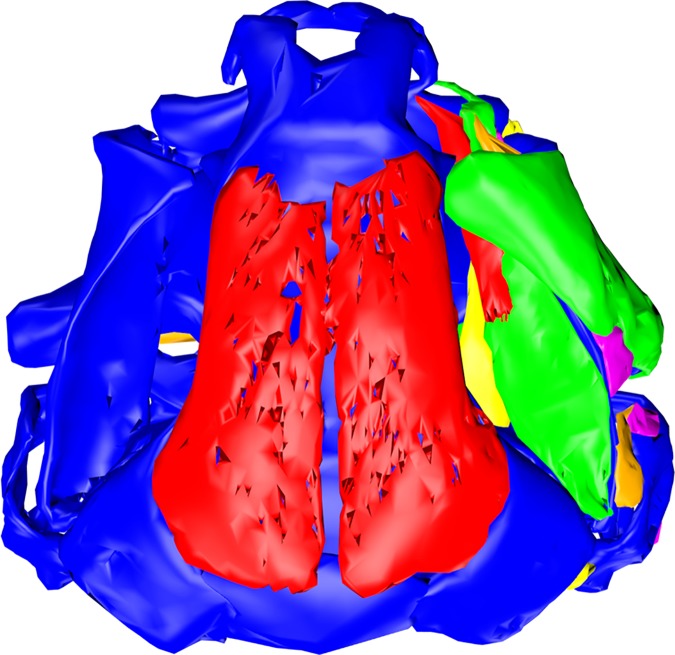
Chondrocranial muscles of *Ranitomeya amazonica*. The chondrocranium (blue) is shown in a dorsal view with all cranial muscles. The reconstruction was done from a stage 41 tadpole (ZFMK 97366). For further information on the muscles we provide a 3D pdf in S3 3D pdf. In this model all muscles can be added to or removed from the scene by a checkbox. The model is rotatable and can be seen in all perspectives.

**Fig 10:**
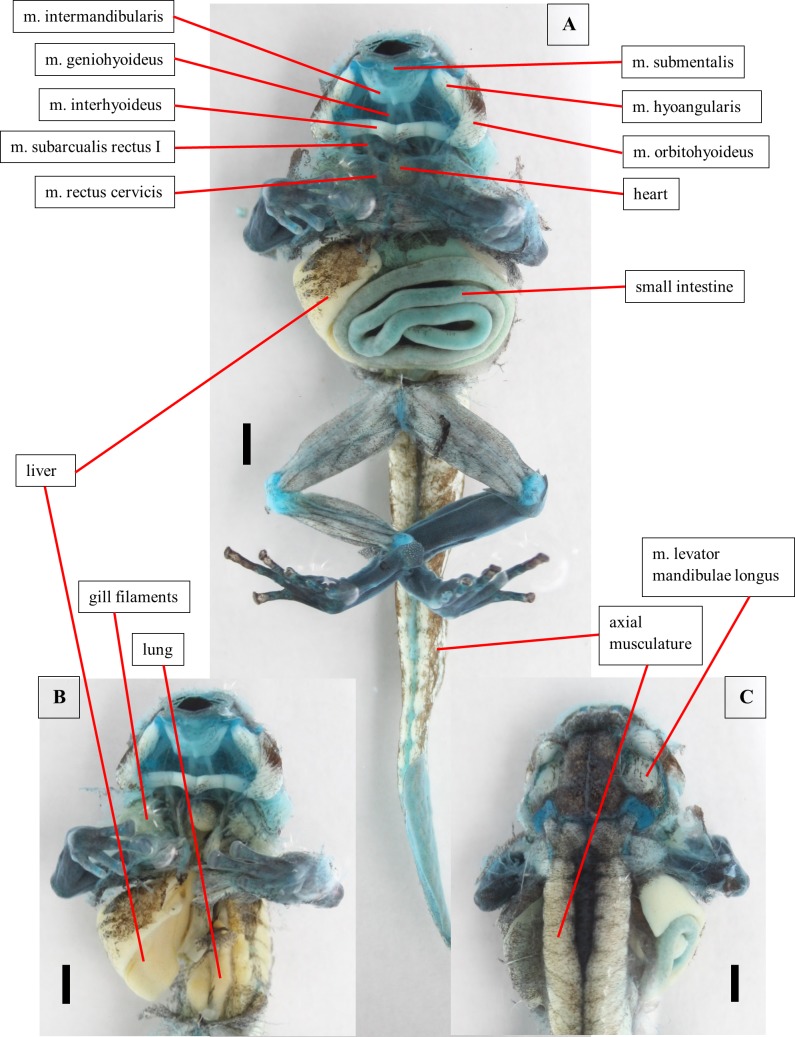
Dissection of a tadpole of *Ranitomeya amazonica*. Dissection of a stage 41 tadpole (ZFMK 97362). Cartilage is stained with alcian blue. (A) Ventral view, body cavity opened, skin removed. (B) Ventral view, intestine removed. (C) Dorsal view, eyes removed. Scale bars 1mm.

Concerning the cranial ossifications the giant, dome-like frontoparietals have to be mentioned. In *R*. *amazonica* these bony plates reach the tectum synoticum, completely covering the trabecula cranii (Fig 8B and 8C). The *m*. *hyoangularis* runs in close association with the *m*. *suspensorioangularis* for all the way from origin to insertion (S3 3D pdf). In *R*. *amazonica* some fibers of the *m*. *orbitohyoideus* originate from the processus antorbitalis. This processus reaches close to the processus muscularis of the palatoquadratum which is the origin of all other fibers of the *m*. *orbitohyoideus* (Fig 9, S3 3D pdf). The two portions of the *m*. *subarcualis rectus I* are separated in *R*. *amazonica* (S3 3D pdf). The *m*. *subarcualis rectus II-IV* of *R*. *amazonica* originates from the basal ceratobranchiale IV. In inserts on the ceratobranchiale III (S3 3D pdf). Its fibers seem to be somehow confluent with the *m*. *constrictor branchialis III* (S3 3D pdf). The origin of the *m*. *rectus cervicis* (continuing as the *m*. *rectus abdominis*) was not found in *R*. *amazonica*. Further differences to the description of *R*. *vanzolinii* are found in the branchial musculature. The *m*. *constr*. *branch*. *II* originates from the basal ceratobranchiale I and inserts on the same gill arch anterior to the terminal commissures (S3 3D pdf). The *m*. *constr*. *branch*. *III* originates from the ceratobranchiale IV and inserts on the terminal commissure of the ceratobranchialia I and II (S3 3D pdf). The *m*. *constr*. *branch*. *IV* inserts on the terminal commissure of the ceratobranchialia II and III (S3 3D pdf). Moreover, the *mm*. *levatores arcuum branchialium I* and *II* were identified in *R*. *amazonica*. They share a common origin on the dorsolateral curvatura posterior quadrati. The *m*. *lev*. *arc*. *branch*. *I* inserts on the anterior ceratobranchiale I, while the *m*. *lev*. *arc*. *branch*. *II* inserts on the commissura terminalis of the ceratobranchialia I and II (S3 3D pdf). The *m*. *tympanopharyngeus* shares a common origin with the branchial levators III and IV on the lateral capsula auditiva (S3 3D pdf). The origin of the *m*. *diaphragmatobranchialis* could not be identified in *R*. *amazonica*. In *R*. *amazonica* the lower liver lobe is not covered by the intestine (Fig 10). The visible part in a ventral view is much bigger than in *R*. *vanzolinii* and *R*. *imitator*.

### Ranitomeya benedicta

**Fig 11:**
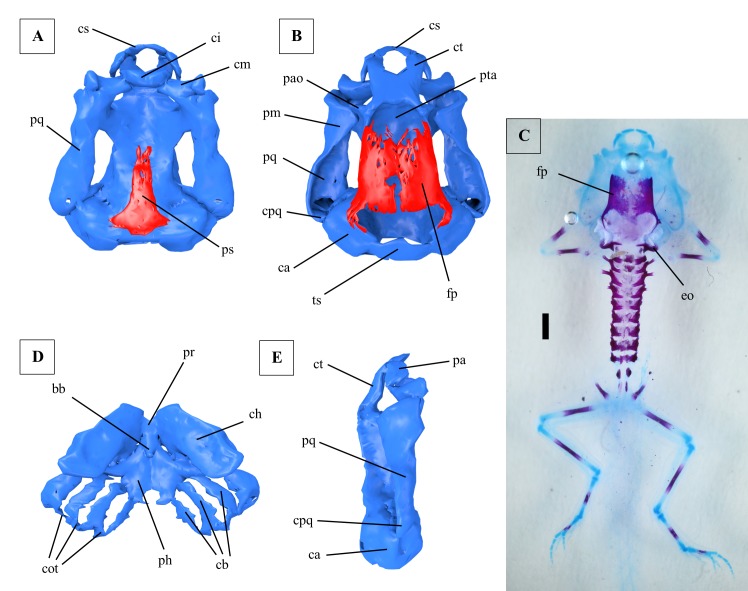
Larval chondrocranium of *Ranitomeya benedicta*. (A) Ventral view, ossifications shown in red, cartilage in blue. (B) Dorsal view, ossifications shown in red, cartilage in blue. (C) Dorsal view, cleared and stained specimen, ossifications stained red, cartilage blue, scale bar 1mm. (D) Hyobranchial apparatus. (E) Lateral view. A, B, D and E were reconstructed from ZFMK 97367. C shows ZFMK 97376. All tadpoles used were in Gosner stage 41. For abbreviations see Fig 2.

**Fig 12:**
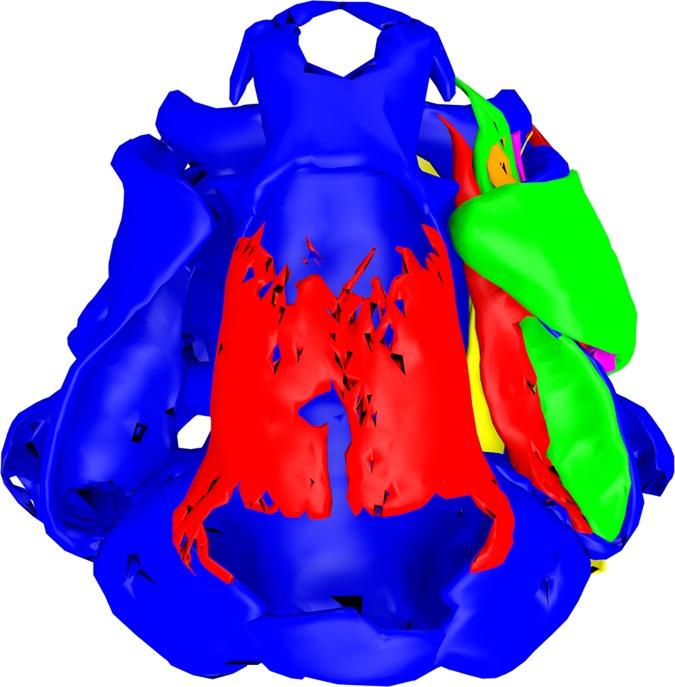
Chondrocranial muscles of *Ranitomeya benedicta*. The chondrocranium (blue) is shown in a dorsal view with all cranial muscles. The reconstruction was done from a stage 41 tadpole (ZFMK 97367). For further information on the muscles we provide a 3D pdf in S4 3D pdf. In this model all muscles can be added to or removed from the scene by a checkbox. The model is rotatable and can be seen in all perspectives.

**Fig 13:**
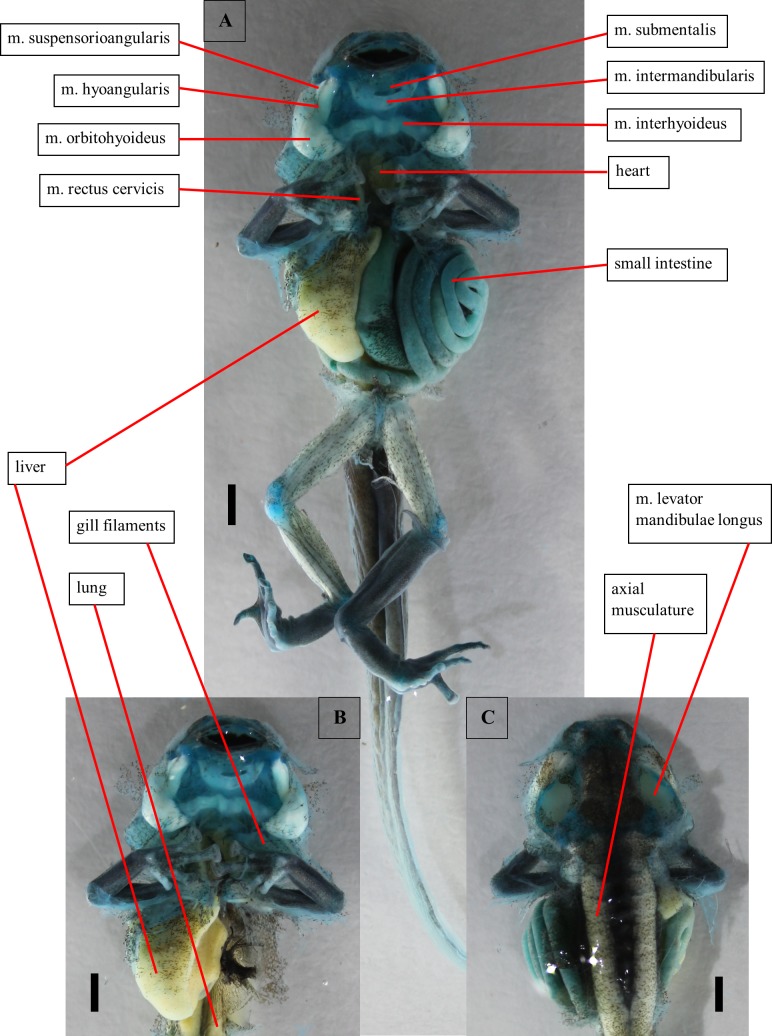
Dissection of a tadpole of *Ranitomeya benedicta*. Dissection of a stage 41 tadpole (ZFMK 97363). Cartilage is stained with alcian blue. (A) Ventral view, body cavity opened, skin removed. (B) Ventral view, intestine removed. (C) Dorsal view, eyes removed. Scale bars 1mm.

The frontoparietals of *R*. *benedicta* (Fig 11B and 11C) are dome-shaped but the braincase is not completely closed. They are bigger than in *R*. *vanzolinii* and *R*. *imitator*, but do not reach the tectum synoticum like in *R*. *amazonica*. In *R*. *benedicta* the origins of the *mm*. *levator mandibulae longus superficialis* and *profundus* can be told apart (Fig 12, S4 3D pdf). Moreover, the insertion points of the *m*. *orbitohyoideus* and the *m*. *suspensoriohyoideus* are distinguishable in *R*. *benedicta* (Fig 12, S4 3D pdf). The *m*. *hyoangularis* runs in close association with the *m*. *suspensorioangularis* for all the way as in *R*. *amazonica* (S4 3D pdf). The *m*. *mandibulolabialis* could not be identified in *R*. *benedicta*. The origins of the two portions of the *m*. *subarcualis rectus I* can be also told apart (S4 3D pdf). As in *R*. *amazonica* the *m*. *subarcualis rectus II-IV* of *R*. *benedicta* originates from the basal ceratobranchiale IV and inserts on the ceratobranchiale III (S4 3D pdf). The *m*. *constrictor branchialis II* originates from the basal ceratobranchiale I and inserts on the same gill arch like in *R*. *amazonica* (S4 3D pdf). The *mm*. *constr*. *branch*. *III* and *IV* were found in the dissection of *R*. *benedicta* but could not be reconstructed from the CT scans. Exact origin and insertion of these muscles are unsure. As a consequence these muscles are not shown in the 3D reconstruction of *R*. *benedicta*. The origin of the *mm*. *levatores arcuum branchialium III* and *IV* is well separated in *R*. *benedicta* (S4 3D pdf). The *m*. *tympanopharyngeus* originates from the posteroventral capsula auditiva and inserts only on oesophagal soft tissue (S4 3D pdf). The origin of the *m*. *diaphragmatobranchialis* could not be identified in *R*. *benedicta*. As in *R*. *amazonica* the lower liver lobe of *R*. *benedicta* is not covered by the intestine in a ventral view (Fig 13).

### Ranitomeya reticulata

**Fig 14:**
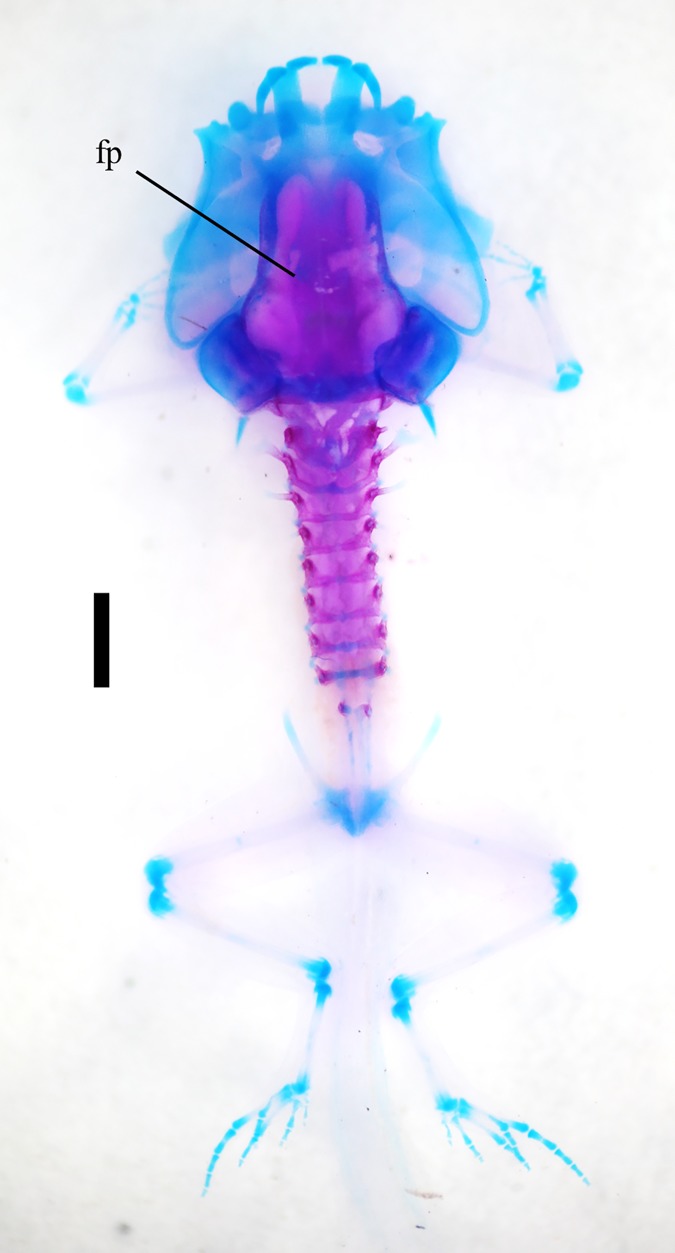
Cleared and stained specimen of *R*. *reticulata*. ZFMK 97378 in a dorsal view, ossifications stained red, cartilage blue, scale bar 1mm. For abbreviations see Fig 2.

**Fig 15:**
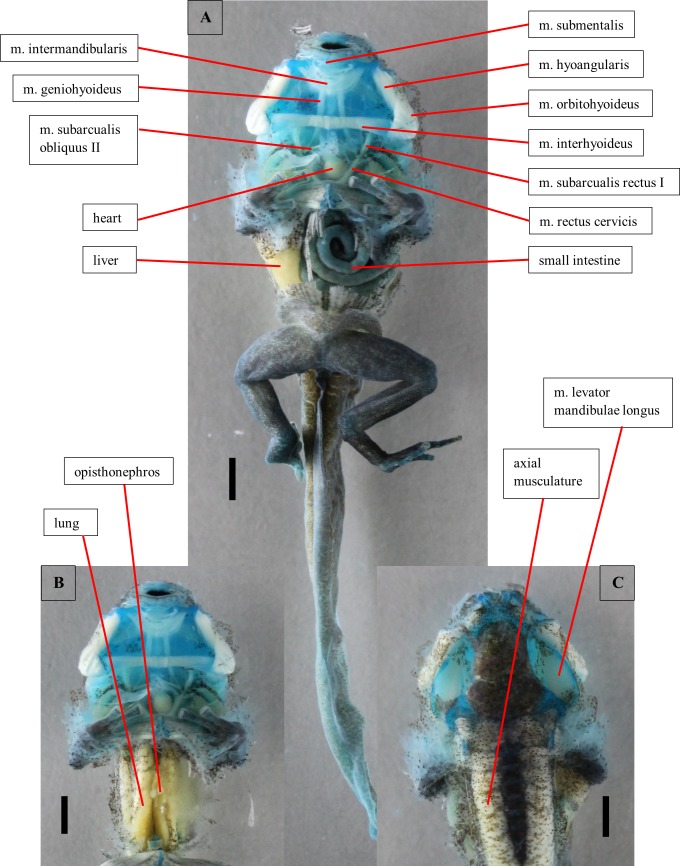
Dissection of a tadpole of *Ranitomeya reticulata*. Dissection of a stage 41 tadpole (ZFMK 97365). Cartilage is stained with alcian blue. (A) Ventral view, body cavity opened, skin removed. (B) Ventral view, intestine removed. (C) Dorsal view, eyes removed. Scale bars 1mm.

The specimen of *R*. *reticulata* used for a micro-CT scan showed an aberrant development and had to be excluded from the study. The cleared and stained specimen shows wide and doming frontoparietals as in *R*. *amazonica* (Fig 14). As in *R*. *amazonica* and *R*. *benedicta* the *m*. *hyoangularis* runs in close association with the *m*. *suspensorioangularis* from its origin up to its insertion. The lower liver lobe is not concealed by the intestine in a ventral view in *R*. *reticulata* (Fig 15).

### Anatomical measurements

The rapid changes in internal anatomy happening in the metamorphic stages 42–46 may influence description of tadpoles that qualify for stage 41 concerning external characters. Nevertheless, we found some stable characters that were differing between the species analyzed in this study: The giant frontoparietals, the *m*. *hyoangularis* ending in line with the *m*. *suspensorioangularis* and the big visible part of the liver in Gosner stage 41 distinguishes *R*. *reticulata*, *R*. *amazonica* and *R*. *benedicta* from *R*. *vanzolinii* and *R*. *imitator* (Table 3). Our specimens of the latter two species show smaller frontoparietals in Gosner stage 41. The *m*. *hyoangularis* separates from the *m*. *suspensorioangularis*. So a cleft between these muscles is formed in stage 41 tadpoles of *R*. *vanzolinii* and *R*. *imitator*. In these specimens just a small part of the liver is visible in a ventral view. In the phylogenetic tree of Brown et al. [2] *R*. *vanzolinii* and *R*. *imitator* are part of the *vanzolinii* species group which is separated from the *reticulata-defleri-amazonica* supergroup that contains *R*. *reticulata*, *R*. *benedicta* and *R*. *amazonica*.

**Table 3:** Anatomical measurements in *Ranitomeya* tadpoles in Gosner stage 41.

	*R*. *vanzolinii*	*R*. *imitator*	*R*. *amazonica*	*R*. *benedicta*	*R*. *reticulata*
Frontoparietal covering of the braincase	• 4% • 11% • 9%	• 15% • 16% • 18%	• 38% • 41% • 38%	• 17% • 21%	• 34% • 29% • 35%
Free running *m*. *hyoangularis*	• 21% • 20%	• 27% • 23% • 17%	• 0% • 0% • 0%	• 3% • 2%	• 0% • 7%
Liver visible	• 13% • 0% • 0%	• 11% • 11% • 1%	• 29% • 26% • 38%	• 32% • 42%	• 32% 30%

Measurements are given as relative values because the tadpoles differed in body size.

### Morphological comparison to other dendrobatids

#### Chondrocranium

Concerning the definition of the Dendrobatidae Haas [20] list two chondrocranial characters: The reduction of tectal cartilages (taenia tecti) and the proximal insertion of the *m*. *rectus cervicis* on the third or fourth branchial arch. Both characters can be clearly confirmed for *Ranitomeya*. Myers [34] calls the absence of the *m*. *levator mandibulae externus superficialis* (as *m*. *adductor mandibulae externus superficialis*) a characterizing feature of dendrobatids. This muscle is also missing in stage 41 tadpoles of *Ranitomeya*.

Haas [35] also analyzed the cranial features of dendrobatid larvae by use of the species *Colostethus nubicola*, *C*. *subpunctatus*, *Dendrobates tinctorius*, *Epipedobates anthonyi*, *E*. *boulengeri*, *E*. *tricolor* and *Phyllobates bicolor*. De Sa and Hill [36] described the chondrocranium of *Dendrobates auratus*. Haas [35] names the quadripartite suprarostral cartilage (independent left and right medial part and pars alaris) a main character shared by all dendrobatids analyzed, but also states that the medial part fuses in advanced states in *Epipedobates* and *Dendrobates*. In our stage 41 *Ranitomeya* tadpoles the four parts are separated by symphyses. Additionally, a small adrostral cartilage was identified in *Epipedobates*, *Phyllobates* and *Colosthetus*. This structure is missing in *Dendrobates* and also in *Ranitomeya*. Furthermore, the curvatura posterior quadrati is highly developed and “expands into the space lateral to the […] capsula auditiva” [35]. Taking into account the figures of the chondrocrania provided by Haas [35] the contact between the curvatura and the capsula in *Phyllobates* and *Dendrobates* is comparable to the character state in *Ranitomeya*. In *Epipedobates* the processus ascendens is still independent and the contact zone is small. At the otic capsule the processus anterolateralis of the crista parotica is present. But only in *Epipedobates* the process is conspicuous. In *Dendrobates* and *Phyllobates* there is just a slight elevation. This character state can be homologized to *Ranitomeya*. All dendrobatids lack a larval otic process. A processus pseudopterygoideus is only conspicuous in *E*. *anthonyi* and tiny or missing in all other species including *Ranitomeya*. Concerning the hyobranchial apparatus the processus anterolateralis of the ceratohyale is an important character. It is present in *Epipedobates* and *Phyllobates* but missing in *Dendrobates* and *Ranitomeya*. The cranial ossifications known from *Ranitomeya* (frontoparietale, prootic, exoccipitale and parasphenoid) are present in all other dendrobatids. The frontoparietals are illustrated for *Dendrobates tinctorius* and *Epipedobates anthonyi* only. In these species they are broad and roofing like in *R*. *amazonica*, *R*. *benedicta*, and *R*. *reticulata*.

The first and second branchial levator, which were not found in *R*. *benedicta*, *R*. *imitator* and *R*. *vanzolinii*, and the *mm*. *interhyoideus posterior* and *diaphragmatopraecordialis*, which could not be found in any species analyzed in here, shall not be treated as phylogenetic characters. Generally cross sectioning and staining allows better identification of tiny and slender muscles [37]. These muscles may be present in the *Ranitomeya* species but were not identified.

There were no concrete chondrocranial differences between the genera *Ranitomeya* and *Dendrobates* detected based on this study and the papers of Haas [35] and De Sa and Hill [36]. Many differences can be found in the genus *Epipedobates* (Table 4). This points on a closer relationship of *Ranitomeya* and *Dendrobates* to each other than to *Epipedobates* which is in congruence with Clough and Summers [38], Vences et al. [39], Santos et al. [40], Vences et al. [41], Darst and Cannatella [42], Grant [4] and Brown et al. [2] (in many of these publications *Ranitomeya* species are still integrated in the genus *Dendrobates*). *Phyllobates* shares characters with both, the *Ranitomeya-Dendrobates* complex and with *Epibedobates* (Table 4).

**Table 4:** Larval chondrocranial characters of dendrobatid species.

	*Ranitomeya*	*Dendrobates*		*Phyllobates*	*Epipedobates*		
	*spec*.	*auratus*	*tinctorius*	*bicolor*	*boulengeri*	*tricolor*	*anthonyi*
Quadripartite suprarostrals	+	+	+	+	+	+	+
Proximal insertion of *m*. *rectus cervicis* on third or fourth branchial arch	+	+	+	+	+	+	+
Reduction of tectal cartilages	+	+	+	+	+	+	+
*M*. *levator mandibulae externus superficialis* present	-	-	-	-	-	-	-
Curvatura posterior quadrati expanding lateral to capsula auditiva	+	+	+	+	-	-	-
Processus anterolateralis of crista parotica conspicious	-	-	-	-	+	+	+
Processus anterolateralis of ceratohyale present	-	-	-	+	+	+	+
Adrostral cartilage present	-	-	-	+	+	+	+
Processus pseudoterygoideus conspicious	-	-	-	-	-	-	+

Characters coded as present (+) or not present (-).

#### Cranial ossifications

The frontoparietale normally appears as a lateral ossification (sometimes with two ossification centers) which expands anteriorly, posteriorly and medially. Most of this expansion normally happens during metamorphosis or postmetamorphically [13,43–45]. Wide and doming frontoparietals in prometamorphic tadpoles as in *R*. *reticulata*, *R*. *amazonica* and *R*. *benedicta* are found in other Dendrobatidae too [35]. The other ossifications present in prometamorphic *Ranitomeya* tadpoles (parasphenoid, prootic and exoccipital) are normally also present in prometamorphic tadpoles of other dendrobatids [35,36]. Generally all these cranial ossifications are well developed in *Ranitomeya*. In *Ranitomeya* the transition from the longitudinal to the transversal part of the parasphenoid is not sharply angled (typical T-shape) like in other Dendrobatidae [35] but more rounded. In some other dendrobatids additionally an ossified premaxilla or squamosal is found [35].

#### Inner organs

Concerning the inner organs a body cavity dominated by a large liver and a large intestine is shared by most tadpoles of various taxa. The length and subdivision of the intestine varies with feeding habits. For suspension feeders like *Ranitomeya* tadpoles a long intestine is typical. Also the organization inside the body cavity with the manicotto and the liver located on the right body side, the small intestine coiling superficially and sinistrally (in a ventral view) concealing the long intestine, coiling dextrally, and other organs is generally found in anuran tadpoles [12,23,33]. Sánchez [23] analyzed the inner organs of 113 poison dart frog species (Aromobatidae and Dendrobatidae). He defined two different character states concerning the organization of the digestive tract. In some species the coils of the digestive tube conceal all other organs, just the right anterior most part of the liver is visible (found in *Rheobates*, *Anomaloglossus*, *Mannophryne*, *Allobates*, *Silverstoneia*, *Epipedobates*, *Colostethus*, *Ameerga*, *Hyloxalus*, *Phyllobates*, *Minyobates*, *Adinobates*, *Adelphobates* and *Dendrobates*). In other species the coils of the digestive tube are shifted to the left body part and the liver can be clearly detected (found in *Andinobates*, *Ranitomeya*, *Oophaga* and *Dendrobates*). In *R*. *vanzolinii* just the right anterior most part of the liver is visible. The other part is concealed by the gut coils. In *R*. *imitator* a stripe of the liver is visible as thick as a gut coil, but it is still overlaid by the gut (in a ventral view). In *R*. *amazonica*, *R*. *reticulata* and *R*. *benedicta* a big part of the liver is visible. In *R*. *benedicta* the gut coils are additionally remarkably shifted to the left body side. Other differences mentioned by Sánchez [23] like changes in diameter or color and the digestive tube being shorter in the latter named organization (big part of liver visible, gut shifted) cannot be found here. The organization of the inner organs is a variable character. For some genera Sánchez [23] found both character states (*Andinobates*, *Dendrobates*). He also included *Ranitomeya* species in his study. Data on the organization of inner organs is available for *R*. *ventrimatriculata* and *R*. *yavaricola*. Both show a digestive tube shifted to the left body side with a remarkable part of the liver visible. Thus both species would be grouped with *R*. *amazonica*, *R*. *reticulata* and *R*. *benedicta*. Taking into account the phylogeny of Brown et al. [2] a gut shifted to the left body side making big parts of the liver visible could be the plesiomorphic character state. This appears to be reasonable since this character state is also found in most species of *Andinobates*, the sister taxon of *Ranitomeya* [2,23]. Just the lineage including *R*. *imitator* and *R*. *vanzolinii* may have changed to a gut concealing all other organs.

### Morphological comparison to bufonids

Following the phylogenetic hypothesis of Pyron and Wiens [46] bufonids are the sister group of dendrobatids. Tadpoles of the bufonid genus *Chaunus* (synonyms *Rhinella* and *Bufo*; tadpoles belonging to *C*. *arenarum* and *C*. *spinulosus*) were examined by Vera Candioti ([12, based on [20,47]). Larson [48] described the larval chondrocranium of *Bufo americanus*. The otic capsules of *Chaunus* show an acute anterolateral process, less developed in *Bufo*. This process is just a slight elevation in the *Ranitomeya* species examined in here. A quadratoorbital commissure, a slender cartilaginous bar, which interconnects the processus muscularis to the braincase, is found in *Chaunus* and *Bufo*. It meets the braincase at the position of the processus antorbitalis. In *Ranitomeya* the muscular process reaches close to the processus antorbitalis. In *Chaunus* and *Bufo* the palatoquadrate just forms a slight curvatura posterior quadrati and its posterior end does not reach the capsula auditiva. The anterior aspect of the ceratohyale of *Chaunus* and *Bufo* is dominated by big processes, additionally to the condylus: the anterior and the anterolateral process of the ceratohyale. In *Ranitomeya* the anterior process is flat and rounded, the anterolateral process is missing completely. Tectal cartilages are missing in *Ranitomeya*, but found in *Chaunus spinulosus* and *Bufo americanus*. The *m*. *levator mandibulae longus* originates from the posterolateral palatoquadrate and the lateral ascending process because a strong curvatura is missing in *Chaunus*. The *m*. *lev*. *mand*. *internus* originates from the ventral ascending process, not from the capsula auditiva like in *Ranitomeya*. The *mm*. *lev*. *mand*. *externus superficialis* and *lateralis* are present in *Chaunus*. The *m*. *subarcualis rectus I* of *Chaunus* has a third insertion on the second gill arch ([12], based on [20,47 and 48]).

In summary, many differences between the larval chondrocrania of bufonids and *Ranitomeya* are listed but the general shape of the chondrocrania is similar. Haas [20] lists the “clearly concave [curvatura posterior quadrati] with bulging and pronounced margin” a synapomorphy of bufonids and dendrobatids. As described here the highly concave curvatura of *Ranitomeya* that is connected to the otic capsule is still a clearly different character state. On the other hand this curvatura is also less pronounced in other dendrobatids [35]. Additionally some processes are reduced or missing in *Ranitomeya* (processus anterolateralis of capsula auditiva and processus anterolateralis of ceratohyale), but are conspicuous in other dendrobatids [35]. Their presences in bufonids might be a plesiomorphic character state [20]. The quadratoorbital commissure of bufonids and the towering processus muscularis of *Ranitomeya*, reaching close to the processus antorbitalis might be treated as similar character states.

## Supporting information

S1 3D pdf Chondrocranial muscles of *Ranitomeya vanzolini*. The chondrocranium (blue) is shown with all cranial muscles. The reconstruction was done from a stage 41 tadpole (ZFMK 97369). In this model all muscles can be added to or removed from the scene by a checkbox. The model is rotatable and can be seen in all perspectives. Zooming in and out is possible to. For muscles sharing a common origin or insertion the respective contact point to the chondrocranium is reconstructed only for one muscle. For the *m*. *suspensorioangularis* and the *m*. *hyoangularis* we know that they have no common insertion but insert side by side (see Fig 4). In the reconstruction the two insertion points could not be told apart.
(PDF)

S2 3D pdf Chondrocranial muscles of *Ranitomeya imitator*. The chondrocranium (blue) is shown with all cranial muscles. The reconstruction was done from a stage 41 tadpole (ZFMK 97368). In this model all muscles can be added to or removed from the scene by a checkbox. The model is rotatable and can be seen in all perspectives. Zooming in and out is possible to. For muscles sharing a common origin or insertion the respective contact point to the chondrocranium is reconstructed only for one muscle. For the *m*. *suspensorioangularis* and the *m*. *hyoangularis* we know that they have no common insertion but insert side by side (see Fig 7). In the reconstruction the two insertion points could not be told apart.
(PDF)

S3 3D pdf Chondrocranial muscles of *Ranitomeya amazonica*. The chondrocranium (blue) is shown with all cranial muscles. The reconstruction was done from a stage 41 tadpole (ZFMK 97366). In this model all muscles can be added to or removed from the scene by a checkbox. The model is rotatable and can be seen in all perspectives. Zooming in and out is possible to. For muscles sharing a common origin or insertion the respective contact point to the chondrocranium is reconstructed only for one muscle. For the *m*. *suspensorioangularis* and the *m*. *hyoangularis* we know that they have no common insertion but insert side by side (see Fig 10). In the reconstruction the two insertion points could not be told apart.
(PDF)

S4 3D pdf Chondrocranial muscles of *Ranitomeya benedicta*. The chondrocranium (blue) is shown with all cranial muscles. The reconstruction was done from a stage 41 tadpole (ZFMK 97367). In this model all muscles can be added to or removed from the scene by a checkbox. The model is rotatable and can be seen in all perspectives. Zooming in and out is possible to. For muscles sharing a common origin or insertion the respective contact point to the chondrocranium is reconstructed only for one muscle. For the *m*. *suspensorioangularis* and the *m*. *hyoangularis* we know that they have no common insertion but insert side by side (see Fig 13). In the reconstruction the two insertion points could not be told apart.
(PDF)

S1 Table Larval cranial muscles of *Ranitomeya* species with origin and insertion. (DOCX)
