# Supplementary material for: Morphological comparison of five species of poison dart frogs of the genus Ranitomeya (Anura: Dendrobatidae) including the skeleton, the muscle system and inner organs
Source: PLoS One. 2017 Feb 24;12(2):e0171669. doi: 10.1371/journal.pone.0171669 (PMC5325227; doi:10.1371/journal.pone.0171669)
Supplement: S1 Table — (DOCX) [file pone.0171669.s005.docx]

| **Musculus** |  | **RV** | **RI** | **RA** | **RB** | **Comment** |
| --- | --- | --- | --- | --- | --- | --- |
| Levator mandibulae longus superficialis | Origin | Curvatura posterior quadrati | | | | Shares origin with m. levator mandibulae longus profundus in RV and RA. |
|  | Insertion | Dorsoposterior cartilago meckeli | | | |  |
| Levator mandibulae longus profundus | Origin | Curvatura posterior quadrati | | | | Shares origin with m. levator mandibulae longus superficialis in RV and RA.  Shares insertion with m. levator mandibulae externus in RV, RI, RA and RB. |
|  | Insertion | Ventroposterolateral cartilago suprarostralis | | | |  |
| Levator mandibulae externus superficialis | Origin | Absent | | | | Absent. |
|  | Insertion |  |  |  |  |  |
| Levator mandibulae externus profundus | Origin | Inner side of palatoquadratum | | | | Shares insertion with m. levator mandibulae longus profundus in RV, RI, RA and RB. |
|  | Insertion | Ventroposterolateral cartilago suprarostralis | | | |  |
| Levator mandibulae articularis | Origin | Inner side of palatoquadratum | | | |  |
|  | Insertion | Dorsolateral cartilago meckeli | | | |  |
| Levator mandibulae internus | Origin | Ventral capsula auditiva | | | |  |
|  | Insertion | Posterolateral cartilago meckeli | | | |  |
| Levator mandibulae lateralis | Origin | Absent in the studied stage | | | | Absent in the studied stage. |
|  | Insertion |  |  |  |  |  |

| **Musculus** |  | **RV** | **RI** | **RA** | **RB** | **Comment** |
| --- | --- | --- | --- | --- | --- | --- |
| Quadrato-angularis | Origin | Ventral palatoquadratum | | | |  |
|  | Insertion | Retroarticular process of cartilago meckeli | | | |  |
| Hyoangularis | Origin | Ventrolateral ceratohyale | | | | Shares insertion with m. suspensorioangularis in RV, RI, RA and RB. |
|  | Insertion | Retroarticular process of cartilago meckeli | | | |  |
| Suspensorio-angularis | Origin | Dorsal and posterolateral palato-quadratum | Dorsal and posterolateral palato-quadratum (two heads) / alimentary canal (one head) | Lateral and posterolateral palato-quadratum | Dorsal and posterolateral palato-quadratum | Shares insertion with m. hyoangularis in RV, RI, RA and RB.  Originates with three heads in RI. |
|  | Insertion | Retroarticular process of cartilago meckeli | | | |  |
| Orbito-hyoideus | Origin | Processus muscularis of palatoquadratum | | Processus muscularis of palato-quadratum / posterior processus antorbitalis | Processus muscularis of palato-quadratum | Shares insertion with m. suspensoriohyoideus in RV, RI and RA**.** |
|  | Insertion | Posteroventral ceratohyale | | | |  |
| Suspensorio-hyoideus | Origin | Dorsolateral palatoquadratum | | | | Shares insertion with m. orbitohyoideus in RV, RI and RA. |
|  | Insertion | Posteroventral ceratohyale | | | |  |

| **Musculus** |  | **RV** | **RI** | **RA** | **RB** | **Comment** |
| --- | --- | --- | --- | --- | --- | --- |
| Submentalis | Origin | Ventral cartilago infrarostralis | | | | Not shown in 3D reconstructions. |
|  | Insertion | Arching from one side to the other | | | |  |
| Mandibulo-labialis | Origin | Cartilago meckeli | | | ? | Not shown in 3D reconstructions.  Not identified in RB. |
|  | Insertion | Lateral oral disc | | | ? |  |
| Inter-mandibularis | Origin | Median raphe | | | |  |
|  | Insertion | Anteroventral cartilago meckeli | | | |  |
| Interhyoideus | Origin | Median raphe | | | |  |
|  | Insertion | Ventral ceratohyale | | | |  |
| Genio-hyoideus | Origin | Ventral planum hypobranchiale | | | | Two insertions. |
|  | Insertion | Ventral cartilago infrarostralis / soft tissue of glottis | | | |  |

| **Musculus** |  | **RV** | **RI** | **RA** | **RB** | **Comment** |
| --- | --- | --- | --- | --- | --- | --- |
| Subarcualis obliquus II | Origin | Posterior basibranchiale (processus urobranchialis) | | | |  |
|  | Insertion | Ceratobranchiale III | | | |  |
| Subarcualis rectus I (dorsal portion) | Origin | Lateral processus posterior hyalis | | | | Shares origin with ventral portion in RV. |
|  | Insertion | Ceratobranchiale I | | | |  |
| Subarcualis rectus I (ventral portion) | Origin | Lateral processus posterior hyalis | | | | Shares origin with dorsal portion in RV. |
|  | Insertion | Ceratobranchiale III | | | |  |
| Subarcualis rectus II-IV | Origin | Basal ceratobranchiale III or IV | | Basal ceratobranchiale IV | | Not shown in 3D reconstructions of RV and RI. |
|  | Insertion | Ceratobranchiale II | | Ceratobranchiale III | |  |
| Rectus cervicis | Origin | Abdominal wall (as m. rectus abdominis) | | ? | | Continues as m. rectus abdominis. |
|  | Insertion | Ceratobranchiale III | | | |  |

| **Musculus** |  | **RV** | **RI** | **RA** | **RB** | **Comment** |
| --- | --- | --- | --- | --- | --- | --- |
| Constrictor branchialis I | Origin | Absent | | | | Absent. |
|  | Insertion |  |  |  |  |  |
| Constrictor branchialis II | Origin | Basal ceratobranchiale II | | Basal ceratobranchiale I | |  |
|  | Insertion | Commissura terminalis of cerato-branchialia I and II | | Ceratobranchiale I | |  |
| Constrictor branchialis III | Origin | Basal ceratobranchiale III | | | ? | Present in RB but origin and insertion not identified.  Not shown in 3D reconstructions of RB. |
|  | Insertion | Terminal ceratobranchiale II | | Commissura terminalis of cerato-  branchialia I and II | ? |  |
| Constrictor branchialis IV | Origin | Ceratobranchiale III | | | ? | Present in RB but origin and insertion not identified.  Not shown in 3D reconstructions of RB. |
|  | Insertion | Terminal ceratobranchiale III | | Commissura terminalis of cerato-branchialia II and III | ? |  |

| **Musculus** |  | **RV** | **RI** | **RA** | **RB** | **Comment** |
| --- | --- | --- | --- | --- | --- | --- |
| Levator arcuum branchialium I | Origin | ? | ? | Dorsolateral curvatura posterior quadrati | ? | Not identified in RV, RI and RB.  Shares origin with m. levator arcuum branchialium II in RA. |
|  | Insertion | ? | ? | Anterior cerato-branchiale I | ? |  |
| Levator arcuum branchialium II | Origin | ? | ? | Dorsolateral curvatura posterior quadrati | ? | Not identified in RV, RI and RB.  Shares origin with m. levator arcuum branchialium I in RA. |
|  | Insertion | ? | ? | Commissura terminalis of cerato-branchialia I and II | ? |  |
| Levator arcuum branchialium III | Origin | Lateral capsula auditiva | | | | Shares origin with m. levator arcuum branchialium IV in RV and RA.  Shares origin with m. tympanopharyngeus in RA. |
|  | Insertion | Commissura terminalis of ceratobranchialia II and III | | | |  |
| Levator arcuum branchialium IV | Origin | Lateral capsula auditiva | | | | Shares origin with m. levator arcuum branchialium III in RV and RA.  Shares origin with m. tympanopharyngeus in RA. |
|  | Insertion | Posterior ceratobranchiale IV | | | |  |
| Tympano-pharyngeus | Origin | Posterior capsula auditiva | Posteroventral capsula auditiva | Lateral capsula auditiva | Posteroventral capsula auditiva | Shares origin with mm. levatores arcuum branchialium III and IV in RA. |
|  | Insertion | Oesophagal and pericardial soft tissue | | | Oesophagal soft tissue |  |

| **Musculus** |  | **RV** | **RI** | **RA** | **RB** | **Comment** |
| --- | --- | --- | --- | --- | --- | --- |
| Interhyoideus posterior | Origin | ? | | | | Not identified. |
|  | Insertion | ? | | | |  |
| Dia-phragmato-praecordialis | Origin | ? | | | | Not identified. |
|  | Insertion | ? | | | |  |
| Dia-phragmato-branchialis | Origin | Abdominal wall | ? | ? | | Not identified in RI.  Origin unsure in RA and RB. |
|  | Insertion | Commissura terminalis of cerato-branchialia III and IV | ? | Commissura terminalis of ceratobranchialia III and IV | |  |
